# Perceived Medical School stress of undergraduate medical students predicts academic performance: an observational study

**DOI:** 10.1186/s12909-017-1091-0

**Published:** 2017-12-16

**Authors:** Thomas Kötter, Josefin Wagner, Linda Brüheim, Edgar Voltmer

**Affiliations:** 1 Institute of Social Medicine and Epidemiology, University of Lübeck, Ratzeburger Allee 160, 23562 Lübeck, Germany; 2 Division of Teaching and Learning, Lübeck Medical School, University of Lübeck, Ratzeburger Allee 160, 23562 Lübeck, Germany; 3 Department of Quality Management and Organizational Development, University of Lübeck, Ratzeburger Allee 160, 23562 Lübeck, Germany; 4 Chair of Health Sciences, Friedensau Adventist University, An der Ihle 19, 39291 Möckern-Friedensau, Germany

**Keywords:** Education, Medical, Undergraduate; Students, Medical; Stress, Psychological, Questionnaires and surveys, Assessment, Educational

## Abstract

**Background:**

Medical students are exposed to high amounts of stress. Stress and poor academic performance can become part of a vicious circle. In order to counteract this circularity, it seems important to better understand the relationship between stress and performance during medical education. The most widespread stress questionnaire designed for use in Medical School is the “Perceived Medical School Stress Instrument” (PMSS). It addresses a wide range of stressors, including workload, competition, social isolation and financial worries. Our aim was to examine the relation between the perceived Medical School stress of undergraduate medical students and academic performance.

**Methods:**

We measured Medical School stress using the PMSS at two different time points (at the end of freshman year and at the end of sophomore year) and matched stress scores together with age and gender to the first medical examination (M1) grade of the students (*n* = 456).

**Results:**

PMSS scores from 2 and 14 months before M1 proved to be significant predictors for medical students’ M1 grade. Age and gender also predict academic performance, making older female students with high stress scores a potential risk group for entering the vicious circle of stress and poor academic performance.

**Conclusions:**

PMSS sum scores 2 and 14 months before the M1 exam seem to have an independent predictive validity for medical students’ M1 grade. More research is needed to identify potential confounders.

**Electronic supplementary material:**

The online version of this article (doi: 10.1186/s12909-017-1091-0) contains supplementary material, which is available to authorized users.

## Background

Medical students are exposed to a high amount of stress during their training [1, 2]. Specific Medical School stressors have been identified, including exposure to death and human suffering, the highly competitive environment and ethical conflicts [3]. Stress and poor academic performance can become part of a vicious circle (increasing stress leads to decreasing performance, which, in turn, increases stress) in the course of medical education [3] with a potential negative impact on students’ and physicians’ health and, potentially, the quality of patient care [4]. In order to counteract this circularity, it seems important to better understand the relationship between stress and performance during medical education. This relationship has been the subject of earlier studies [5, 6]. However, to our knowledge, it has never been investigated using an instrument which addresses specific Medical School stressors.

Measuring specific Medical School stress is, however, important; not only does it allow the further investigation of sources of stress and starting points for health-promoting interventions, but it is also necessary in evaluating such interventions for medical students.

To date, the most widespread validated stress questionnaire designed for use in Medical School is the “Perceived Medical School Stress Instrument” (PMSS) introduced by Vitaliano et al. [7]. It addresses a wide range of possible stressors, including workload, competition, social isolation and financial worries, and has been used in a number of cross-sectional [8, 9], longitudinal [10, 11] and interventional studies [12, 13]. PMSS scores predict mental health problems after graduation [14]. In addition to the original US version, the PMSS instrument has been translated into and adapted to the Norwegian [15], German [16], and Korean languages [17] and has been used in the respective countries. A variety of other, more general, instruments has been used in earlier studies on medical students stress. Unfortunately, the heterogeneity of these studies prohibits direct comparisons of their results. Consequently, the use of standardized measures is called for [18]. The authors of a recent systematic review of stress-management programs for medical students conclude that the PMSS should be among the standard set of outcome measures for this area of research [19].

In this study, our aim was to examine, to our knowledge for the first time, the relation between the perceived Medical School stress of undergraduate students, measured using the PMSS, and academic performance (first medical examination [M1] grade). We hypothesized that perceived Medical School stress would prove to be a predictor of the M1 grade and that students with lower perceived stress levels would perform better.

## Methods

### Study design

For the present study, we used data from an ongoing prospective, longitudinal, observational study on medical students’ health, the Lübeck University Students Trial (LUST) [20, 21].

### Setting

The study was conducted at Lübeck Medical School, a section of the public University of Lübeck. About 1500 students are enrolled in the medical study program and each year, and about 185 freshmen are admitted to Lübeck Medical School.

### Participants

We included data from students who passed their M1 between August 2013 and August 2015 and at the same time took part in the above mentioned LUST Study.

### Outcome

As for the outcome, we chose the grade of the written M1, which is a nationwide standardized, two-day, 320 questions exam. M1 takes part twice each year, in March and August. In order to pass the exam, students have to also pass an oral exam in certain subjects, which is standardized to a far lesser degree. M1 grades, which range from 1 (best) to 5 (failed), are recorded and archived by the Medical School administration.

The M1 exam can be taken after a minimum of 2 years of Medical School. Therefore, we used data from the classes of 2011 onward. Students have to fulfill certain criteria, e.g. certificates for 14 pre-clinical subjects, in order to be allowed to register for the examination. Passing the M1 exam is a prerequisite for continuing medical education at the clinical stage. At Lübeck Medical School, about 75% of all medical students pass the M1 exam after 2 years, compared to 70% nationwide. In total, about 95% of all medical students pass the M1 exam. The mean duration for pre-clinical studies is 2.3 years.

### Predictors

We measured perceived Medical School stress using the German language version of the Perceived Medical School Stress scale (PMSS-D), which comprises 13 items [16]. Each item can be answered on a five-point Likert scale (1 = I strongly disagree; 5 = I strongly agree). For the original English-language items, see in Additional file 1: Table S1.

Age and gender were surveyed as socio-demographic characteristics of the study participants and as control variables.

Predictors were measured at two different time points (T1 and T2). T1 measures were taken at the end of the freshman year (June 2012 and 2013) and T2 measures at the end of the sophomore year, 2 months before the M1 exam (June 2013 and 2014).

All surveys were conducted using the web-based SurveyMonkey (SurveyMonkey Europe, Dublin, Ireland).

### Preventing selection bias

In order to reduce bias due to non-response, we offer all participants of the LUST study a reward in terms of a book voucher to the amount of 5 Euro per survey.

### Study size

The cohort size was predefined by the size of each class at Lübeck Medical School (*n* = 185). For this study, we used data from three consecutive classes (freshmen of 2011, 2012 and 2013).

### Data management

Outcome data (M1 results) and predictors were imported into IBM SPSS Statistics for Windows Version 22.0 (IBM Corp., Armonk, NY, USA) files from a Microsoft Excel 2010 file (Microsoft Corp., Redmond, WA, USA) and a Microsoft Access 2010 database, respectively. Both files were then matched using the students’ matriculation number as the key variable. This number is used as a pseudonym for LUST and cannot be readily linked to real names by the investigators [20].

### Statistical methods

Data analyses were conducted in IBM SPSS Statistics for Windows Version 22.0. We substituted up to one missing value in the PMSS questions by the mean value of the 12 other items and calculated the sum score (range: 13–65) for further analyses. We then excluded incomplete data sets. We used two-tailed t-tests to compare means of continuous variables and report results as means (*M*) ± standard deviation (*SD*). For gender, data were analysed using a chi-square test and the result reported as a percentage. In order to express bivariate correlations, we used Spearman’s ρ. Effect sizes are reported using Cohen’s d. We considered values of 0.2 small, 0.5 medium and 0.8 large effect sizes [22]. We used linear regression analyses in order to confirm correlations between PMSS scores and M1 grades. We conducted separate regression analyses for both PMSS T1 scores and PMSS T2 scores. We included age and gender in both analyses in order to control for them.

## Results

### Participants

After the exclusion of incomplete data-sets, 386 PMSS T1 scores and 352 PMSS T2 scores could be matched to 456 M1 grades (85 and 77% respectively). Of these, 67% were female and 33% male (there was no missing data for this variable). The gender distribution of the sample resembles that of the whole classes of 2011–13 (*n* = 555, 66% female). Regarding age, the sample (*M* age: 22.3 years at T2; *n* = 5 missing information) is also comparable to the whole classes (*M* age: 22.8 years at T2). In our sample, we observed a two-peak age distribution with about 80% of the students being from 18 to 24 years of age at T2 and 20% of the students being 25 years or older (up to 37 years). This distribution resembles the admission criteria for Medical School in Germany: 80% of all students are admitted using the final school exam grade as the main criterion and 20% of all students are admitted after a waiting time of, currently, about 7 years. The mean age of the “older 20%” was 6 years higher than that for the “younger 80%” in our sample (27.10 ± 1.80 vs 21.13 ± 1.24 years; *t* − 30.11, df 133.745, *p* < .01; 95% CI of the difference 6.37–5.58). The gender distribution in the two age groups did not differ.

The characteristics of the sample are displayed in Table 1.

**Table 1:** Characteristics of the sample

	Male	Female	Overall
*n (%)*	151 (33)	305 (67)	456
*M* Age T2 *(SD)* in years	22.49 (2.52)	22.26 (2.89)	22.33 (2.77)
*M* M1 Grade *(SD)*	2.50 (0.89)	2.90 (0.88)	2.77 (0.91)
*M* PMSS T1 *(SD)*	29.24 (5.64)	29.31 (6.03)	29.28 (5.90)
*M* PMSS T2 *(SD)*	30.96 (6.60)	31.62 (7.13)	31.42 (6.97)

Amongst the freshmen of 2011, 2012 and 2013, of those who did not pass the M1 exam between August 2013 and August 2015 (*n* = 11), 81% were female and the mean age was 24.1 years at T2.

### M1 grades

The mean M1 grades differed between the classes 2011–13 (2011: 2.94 ± 0.86; 2012: 2.72 ± 0.92; 2013: 2.64 ± 0.92). They also differed depending on the time of taking the exam (after four semesters: 2.64 ± 0.87; after more than four semesters: 3.52 ± 0.72). Finally, M1 grades differed between male and female medical students (2.50 vs 2.90; *t* 4.56, df 454, *p* < .01; 95% CI of the difference 0.23–0.58; see Table 1). Cohen’s d for this difference is 0.5 (medium effect size). The mean M1 grade of the “older 20%” of the medical students in our sample was statistically significantly higher when compared to the rest of the sample (3.07 ± 0.84 vs 2.69 ± 0.91; *t* − 3.72, df 454, *p* < .01; 95% CI of the difference 0.58–0.18).

### PMSS (T1 & T2)

Both T1 and T2 PMSS scores did not differ between male and female medical students (see Table 1). However, we observed a statistically significant increase in Medical School stress as measured by the PMSS between T1 and T2 (29.10 to 31.58, *t* − 8.09, df 329, *p* < .01, 95% CI of the difference 1.87–3.08, *n* = 330). Cohen’s d for this increase is 0.5 (medium effect size). We saw statistically significant increases in all but four possible stressors covered by the PMSS (see in Additional file 1: Table S2). The mean scores for *uncertainty of the expectations by the faculty* and *lack of support from the administration* increased numerically, the difference not being statistically significant. For *financial worries* and *concerns about accommodation*, the mean scores decreased (statistically significant for the former but not for the latter). We also observed positive bivariate correlations between age and both T1 and T2 PMSS scores (Spearman’s ρ: 0.20 for PMSS T1 and 0.22 for PMSS T2 scores, in both cases *p* < .01). The mean PMSS scores of the “older 20%” of the medical students in our sample were statistically significantly higher when compared to the rest of the sample (T1: 31.27 ± 6.14 vs 28.79 ± 5.75; *t* − 3.41, df 388, *p* < .01; 95% CI of the difference 3.91–1.05; T2: 33.96 ± 5.91 vs 30.81 ± 7.04; *t* − 3.50, df 354, *p* < .01; 95% CI of the difference 4.92–1.38). The mean PMSS scores of the freshmen of 2011, 2012 and 2013 who did not pass the M1 exam between August 2013 and August 2015 were 31.00 ± 7.53 (T1) and 32.00 ± 2.00 (T2). The mean M1 score did not differ significantly between those with complete and those with missing / incomplete PMSS data for T1 and / or T2.

### Correlation of Medical School stress & M1 grades

Spearman’s ρ for the bivariate correlation between PMSS T1 and T2 scores and the M1 grade were 0.21 and 0.22, respectively (in both cases *p* < .01).

### Linear regression analyses

The linear regression analyses revealed both PMSS T1 and T2 scores as independent predictors of the M1 grade (see Tables 2 and 3) with better M1 grades for students with lower PMSS scores. Age and gender were also shown to be statistically significant predictors of the M1 grade with better M1 grades for male and younger students.

**Table 2:** Linear regression analysis: Predictors of M1 grade at T1

Predictor	Range	B	95% CI	*p*-Value
Age T1	18–37	.05	.02–.09	< .01
Gender	0 male 1 female	.33	.15–.51	< .01
PMSS T1	0–65	.03	.02–.05	< .01

Nagelkerkes *R* ^*2*^ = .115

**Table 3:** Linear regression analysis: Predictors of M1 grade at T2

Predictor	Range	B	95% CI	*p*-Value
Age T2	18–37	.06	.02–.09	< .01
Gender	0 male 1 female	.30	.11–.49	< .01
PMSS T2	0–65	.02	.01–.03	< .01

Nagelkerkes *R* ^*2*^ = .095

## Discussion

In our prospective, longitudinal study, we linked PMSS scores measured at the end of the freshman and the sophomore year of medical education to the results of the first medical exam. Additionally, gender and age proved to be significant predictors of the M1 grade.

Previous studies investigating the link between stress and academic performance in medical students (using other instruments for stress measurement) showed similar results [5, 23, 24]. Linn and Zeppa [23] point out that the quality of stress is important for its association with academic performance: Students with unfavorable stress showed impaired academic performance, whereas favorable stress was not negatively associated with performance. Stewart et al. conclude, that reported stress levels during pre-clinical education predicted performance in the first 2 years of Medical School. However, the predictive value of stress on performance decreased once pre-Medical-School performance was statistically controlled. Sohail [24] combined quantitative methods linking stress and performance of first year medical students with in-depth interviews exploring sources of stress and relevant coping strategies. Interestingly, the *number* of stress sources correlated slightly more strongly with academic performance as compared to the *level* of stress in Sohail’s study. Despite different measurement and analysis methods, our findings seem to be readily consistent with the existing literature: perceived stress is closely linked to academic performance in early medical education.

To our knowledge, our study is the first to show a statistically significant and relevant difference in academic performance between male and female German medical students (similar results have recently been found for medical science students in Oxford [25]). This finding is quite surprising and not readily explainable, since in Germany, female, in comparison to male, students have better final high school exam grades [26]. Final high school exam grades are, in turn, amongst the best predictors for academic success in Medical School [27]. One explanation for our finding might be that those male students who were admitted to Medical School must have had final high school exam grades at least equal to those of their female peers. And the learning patterns which lead to being more successful than other male high school graduates might prove a good prerequisite for succeeding in Medical School.

The correlation between age and PMSS sum scores at T1 and T2 observed in our study was statistically significant, although rather weak. Yet, older medical students are known to have a number of problems that younger students do not have, such as financial problems and a lack of social integration [28, 29] and, in some cases, difficulties in developing effective study habits due to long periods between high school and admission to Medical School. Our finding that age also predicts the M1 grade is, therefore, less surprising. The ability to cope with higher levels of stress and bounce back might be a mediator between age and academic performance.

We observed an increase in the mean PMSS score between T1 and T2 of about 2.5. This corresponds to a medium effect size, which we interpret as clinically relevant in comparison with the absolute changes achieved in interventional studies aiming to reduce Medical School related stress [13, 30]. A closer look at the different stressors revealed that stress concerning the financial and housing situation of the students decreased between the end of the freshmen and the end of the sophomore year. This finding seems plausible and might reflect a positive adjustment to the changes in life experienced when entering medical school. However, all other aspects of stress covered by the PMSS increased during this time period, making it a potential time window for stress management interventions.

Our analysis of demographic characteristics and the PMSS scores of those study participants, who did not pass the M1 - although numerically few - support our findings: They were older at t2, more likely to be female and had higher mean PMSS scores at T1 and T2.

### Strengths & limitations

This study has several strengths and limitations. To our knowledge, this is the first study linking perceived Medical School stress measured by the PMSS to academic performance. Further strengths of our study are its longitudinal design and the high response rate. The former allowed us to analyze the predictive value of the PMSS score more than 1 year before the M1 exam. At this stage, students with high PMSS scores still have sufficient time to learn and practice stress-management techniques. The high response rate makes any selection bias unlikely.

Due to the limited sample-size, the analyses were not powered to control for multiple potential confounders. Physical and mental health, as well as pre-Medical-School academic performance, could have an influence both on the predictor and the outcome. However, we did control for age and gender as perhaps the most important potential confounders for both T1 and T2 PMSS sum scores.

The single-centred nature of our study may limit the generalizability of the results. However, the age (22.3 vs 21.9 years) and gender (67 vs 70% female) distributions, as well as the mean M1 grades (2.77 vs 2.65), of our study resemble nationwide distributions [31, 32]. The local findings may thus be generalized at least at a national level.

### Implications for research & practice

Our findings might help identify students at risk of poor academic performance relatively early in medical education. Tailored support programs for these particular students may prevent them from getting into a vicious circle of high perceived stress and poor academic performance or help them to break this circle. The PMSS seems to be a helpful instrument for assessing medical students at risk, having especially identified older students in our study.

The predictive value of the PMSS for academic performance needs to be confirmed in further studies. Larger studies would bear the opportunity to control for more potential confounders. In the long run, interventional studies should be conducted in order to show the effects of tailored stress-management interventions on not only perceived Medical School stress but also on academic performance. To further ensure the validity of the PMSS, studies including objective stress measures, e.g. saliva or blood cortisol measurement, could be of use.

Future studies should also examine the relationship between gender and academic performance in Medical School. These studies should take a closer look at mediators between gender and academic performance, e.g. coping strategies and social factors. Qualitative approaches could be helpful for exploring the link between stress and academic performance more closely.

## Conclusion

PMSS sum scores 2 and 14 months before the M1 exam seem to have an independent predictive validity for medical students’ M1 grade. Age, as well as gender, also predicted academic performance in our sample, making older female students with high perceived Medical School stress a potential risk group for entering the vicious circle of stress and poor academic performance. More research, including qualitative approaches, is needed to explore the influence of potential confounders.

## Additional file

Additional file 1: Table S1. PMSS items (english wording); **Table S2.** Longitudinal results from our sample. Description of data / legend: The (english language) wording of the PMSS items [8, 16] is presented in Table S1. Table S2 shows the mean scores for the single PMSS items, as well as the sum scores for T1 and T2. Results of the dependent t-tests for paired samples are also shown in Table S2. (DOCX 18 kb)

## Abbreviations

LUST: Lübeck University Students Trial
M1: First medical examination
PMSS: Perceived Medical School Stress Instrument
