# Supplementary material for: Perceived Medical School stress of undergraduate medical students predicts academic performance: an observational study
Source: BMC Med Educ. 2017 Dec 16;17:256. doi: 10.1186/s12909-017-1091-0 (PMC5732510; doi:10.1186/s12909-017-1091-0)
Supplement: Additional file 1: Table S1. — PMSS items (english wording); Table S2. Longitudinal results from our sample. Description of data / legend: The (english language) wording of the PMSS items [8, 16] is presented in Table S1. Table S2 shows the mean scores for the single PMSS items, as well as the sum scores for T1 and T2. Results of the dependent t-tests for paired samples are also shown in Table S2. (DOCX 18 kb) [file 12909_2017_1091_MOESM1_ESM.docx]

**Additional file 1**

Table A1. PMSS items (english wording)

| PMSS item | wording |
| --- | --- |
| 1 | Medical school fosters a sense of anonymity and feelings of isolation among the students. |
| 2 | I am concerned that I will not be able to endure the long hours and responsibilities associated with clinical training and practice. |
| 3 | I do not know what the faculty/administration expect of me. |
| 4 | Medical training controls my life and leaves too little time for other activities. |
| 5 | I am concerned that I will unable to master the entire pool of medical knowledge. |
| 6 | This medical school is fostering a physician role at the expense of one’s personality and interests. |
| 7 | Medical school is more competitive than I expected. |
| 8 | The attitude of too many of the faculty is that students should be subjected to ‘baptism of fire’. |
| 9 | The majority of students feel that success in medical school is in spite of the administration rather than because of it. |
| 10 | Medical school is cold, impersonal and needlessly bureaucratic. |
| 11 | Medical school is more of a threat than a challenge. |
| 12 | Personal finances are a source of concern to me. |
| 13 | Accommodation is a source of concern to me. |

Table A2. Longitudinal results from our sample

| **PMSS item** | **T1 (M [SD])** | **T2 (M [SD])** | ***t*** | **df** | **p** |
| --- | --- | --- | --- | --- | --- |
| **1** | 2.05 (0.88) | 2.18 (0.99) | -2.58 | 329 | .01 |
| **2** | 2.45 (0.82) | 2.60 (0.89) | -3.17 | 329 | < .01 |
| **3** | 2.22 (0.77) | 2.31 (0.83) | -1.73 | 329 | .09 |
| ***4*** | 3.15 (1.05) | 3.47 (0.96) | -5.93 | 329 | < .01 |
| ***5*** | 3.00 (0.95) | 3.49 (0.95) | -8.42 | 328 | < .01 |
| ***6*** | 1.97 (0.82) | 2.33 (1.02) | -6.40 | 328 | < .01 |
| ***7*** | 2.08 (0.95) | 2.23 (1.02) | -2.70 | 327 | < .01 |
| ***8*** | 2.17 (0.86) | 2.54 (1.09) | -6.21 | 326 | < .01 |
| ***9*** | 2.37 (0.87) | 2.48 (0.96) | -1.86 | 326 | .06 |
| ***10*** | 1.78 (0.67) | 1.96 (0.75) | -3.92 | 328 | < .01 |
| ***11*** | 1.74 (0.74) | 2.05 (0.83) | -6.26 | 328 | < .01 |
| ***12*** | 2.23 (1.06) | 2.12 (1.13) | 1.97 | 329 | .05 |
| ***13*** | 1.97 (1.03) | 1.85 (0.98) | 1.87 | 329 | .06 |
| ***Sum score*** | 29.10 (5.96) | 31.58 (7.05) | -8.09 | 329 | < .01 |

The (english language) wording of the PMSS items [8,16] is presented in Table A1. Table A2 shows the mean scores for the single PMSS items, as well as the sum scores for T1 and T2, respectively. Results of the dependent t-tests for paired samples are also shown in Table A2.
